# Identification of Novel Compound Heterozygous *MYO15A* Mutations in Two Chinese Families with Autosomal Recessive Nonsyndromic Hearing Loss

**DOI:** 10.1155/2021/9957712

**Published:** 2021-05-14

**Authors:** Xiao-Hui Wang, Le Xie, Sen Chen, Kai Xu, Xue Bai, Yuan Jin, Yue Qiu, Xiao-Zhou Liu, Yu Sun, Wei-Jia Kong

**Affiliations:** ^1^Department of Otorhinolaryngology, Union Hospital, Tongji Medical College, Huazhong University of Science and Technology, Wuhan 430022, China; ^2^Institute of Otorhinolaryngology, Tongji Medical College, Huazhong University of Science and Technology, 430022 Wuhan, China

## Abstract

Congenital deafness is one of the most common causes of disability in humans, and more than half of cases are caused by genetic factors. Mutations of the *MYO15A* gene are the third most common cause of hereditary hearing loss. Using next-generation sequencing combined with auditory tests, two novel compound heterozygous variants c.2802_2812del/c.5681T>C and c.5681T>C/c.6340G>A in the *MYO15A* gene were identified in probands from two irrelevant Chinese families. Auditory phenotypes of the probands are consistent with the previously reported for recessive variants in the *MYO15A* gene. The two novel variants, c.2802_2812del and c.5681T>C, were identified as deleterious mutations by bioinformatics analysis. Our findings extend the *MYO15A* gene mutation spectrum and provide more information for rapid and precise molecular diagnosis of congenital deafness.

## 1. Introduction

Congenital deafness is one of the most common birth defects, with an incidence of approximately 1.4 per 1000 newborns screened. More than 60% of neonates with congenital deafness can be attributable to genetic factors [1]. Most of these cases are nonsyndromic hearing loss (NSHL), and autosomal recessive inheritance accounts for up to 80% of NSHL [2]. *GJB2*, *SLC26A4*, and *MYO15A* are the top three common genes responsible for hereditary hearing loss [3, 4]. Mutations in the *MYO15A* gene have been found to lead to autosomal recessive nonsyndromic hearing loss 3 (DFNB3) [5], and new mutations of this gene are constantly being detected.

The *MYO15A* gene spans 71 kb of genomic DNA on chromosome 17p11.2. It contains 66 exons and encodes unconventional myosin-XV protein which is composed of 3530 amino acids [6]. The protein it encodes mainly localizes in the tips of mammalian hair cell stereocilia, which plays a crucial role in the development of stereocilia and the formation of normal auditory function [7, 8]. It shares a structural organization consisting of a N-terminal domain, an ATPase motor domain, a neck region with myosin light chain binding, and a globular tail domain [6, 9]. The integrity of a protein plays a crucial role for its function. Therefore, the truncating mutation which is interrupted by a stop codon may lead to a pathogenic protein. Besides, more than 40 missense mutations have been identified in the moto domain of the *MYO15A* gene associated with autosomal recessive nonsyndromic hearing loss (ARNSHL), where the most mutations occur. In the mouse model, the mutation in the motor domain results shorter stereocilia with an abnormal staircase structure which leads to deafness [4].

Hearing screening combined with genetic diagnosis can help us recognize more pathogenic gene mutations. The mutation spectrum of the *MYO15A* gene is highly heterogeneous which refers to two heterozygous variants present in trans configuration within the same genomic region of interest. Through the study of two independent *MYO15A* gene mutant families, we identified two novel compound heterozygous mutations: c.2802_2812del/c.5681T>C and c.5681T>C/c.6340G>A. Besides, the known pathogenic variant of c.6340G>A provides more evidence to deduce the pathogenicity of the first two novel mutations by the verification between pedigrees.

## 2. Materials and Methods

### 2.1. Family Description

**Figure 1:**
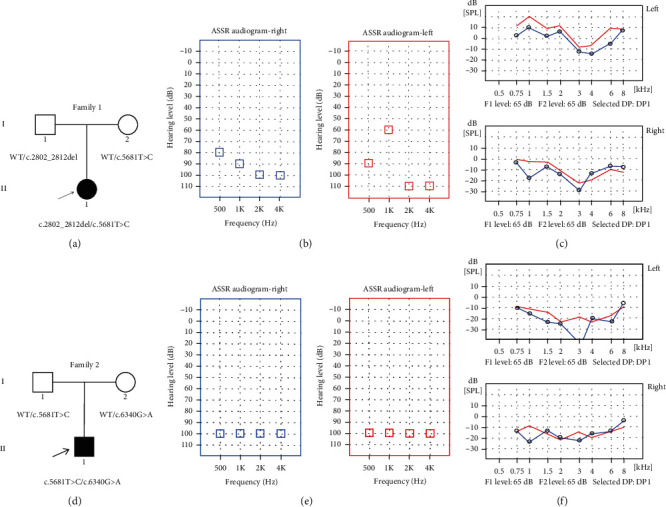
Pedigrees of the affected Family 1 (a) and Family 2 (d) associated with NSHL. The novel compound heterozygous mutations were found in family members. The probands are shown in black. WT: wild type. (b) Auditory steady-state response (ASSR) audiogram of the proband II-1 of Family 1. (c) Distortion product otoacoustic emission (DPOAE) audiogram of both ears of the proband II-1 of Family 1. (e) ASSR audiogram of the proband II-1 of Family 2. (f) DPOAE audiogram of both ears of the proband II-1 of Family 2.

In this study, two Chinese families affected by congenital NSHL were recruited. These two Chinese families contain three family members (daughter/son, mother, and father), respectively. The affected member II-1 of Family 1 (Figure 1(a)): a 2-year-old girl, failed to pass hearing screening and diagnosed with congenital NSHL. The proband II-1 of Family 2 (Figure 1(d)): a 1-year and 5-month-old boy, diagnosed with congenital NSHL as well. Other individuals had no history of hearing impairment.

### 2.2. Clinical Examination

All affected family members underwent clinical evaluation in the Department of Otorhinolaryngology, Union Hospital, Tongji Medical College, Huazhong University of Science and Technology, Wuhan, China. A series of audiological examinations were performed on probands, which included otoscopic examination, behavioral observation audiometry (BOA), auditory immittance, auditory brainstem response (ABR), auditory steady-state evoked response (ASSR), and distortion product otoacoustic emission (DPOAE). The DPOAE were detected in 750, 1000, 1500, 2000, 3000, 4000, 6000, and 8000 Hz frequencies. The degree of hearing loss was defined as mild (26–40 dB HL), moderate (41–55 dB HL), moderately severe (56–70 dB HL), severe (71–90 dB HL), and profound (>90 dB HL). The computed tomography (CT) scan and MRI (Magnetic Resonance Imaging) were also performed on probands. In addition, physical examinations were also performed to rule out other systemic diseases. The probands' parents provided family history and clinical questionnaires, and informed consent was obtained from the whole family for inclusion in the study.

### 2.3. Mutation Detection and Analysis

HDNPL_9957712After obtaining informed consent, 3-5 mL peripheral venous blood samples were obtained from all the family members for NGS+Sanger sequencing. Genomic DNA was extracted from the blood samples according to the manufacturer's standard procedure using the QIAamp DNA Blood Midi Kit (Qiagen Inc., Hilden, Germany). Agarose gel electrophoresis was performed for evaluating the quality and quantity of DNA samples according to the routine protocol. DNA from the proband were performed whole-exome sequencing. Whole-exome capture was performed using the BGI-Exome kit V4 and sequenced by BGI-seq500 with 100 bp paired-end sequencing. Sequenced reads were collected and aligned to the human genome reference (UCSCGRCh37/hg19) by the Burrows-Wheeler Aligner (BWA-MEM, version 0.7.10) [10]. In order to validate the mutations identified in the proband and confirm their cosegregation in the pedigree, DNA from all members of the family was performed Sanger sequenced. After polymerase chain reaction (PCR) amplification and purified the amplified fragments, Sanger sequencing was performed with an ABI3730xl DNA Sequence and the results were analyzed using the Sequencing Analysis 5.2 software (Thermo Fisher Scientific, USA) [11]. The names of variants were referred to the HGVS nomenclature guidelines (http://www.hgvs.org/mutnomen). The methods have been published in our previous studies [12, 13].

### 2.4. Predictions of the Pathogenic Variations

Several different computer algorithms were used to predict the pathogenic features of missense variants: MutationTaster (http://www.mutationtaster.org/), PROVEAN (http://provean.jcvi.org/), and PolyPhen-2 (http://genetics.bwh.harvard.edu/pph2/). The PROVEAN scores indicated deleterious and neutral function, with a cut-off score set at -2.5. The PolyPhen-2 score ranges from 0.0 to 1.0. Variants with scores of 0.0 are predicted to be benign. Values closer to 1.0 are more confidently predicted to be deleterious. Phylogenetic analysis of different sequence alignments was performed by Clustal Omega (https://www.ebi.ac.uk/Tools/msa/clustalo/). The orthologous *MYO15A* protein sequences include mouse, chimpanzee, rat, and macaque. The mutations were detected in the 1000 Genomes database, the dbSNP database, and the Exome Aggregation Consortium database (ExAC). Pathogenicity of the variants was classified based on the guidelines of ACMG 2015 [14].

## 3. Results

### 3.1. Clinical Data

#### 3.1.1. Family 1

The proband II-1 of Family 1 test results were as follows. BOA showed poor hearing, and acoustic immittance test results show that the tympanograms were type As (left ear) and type A (right ear). The thresholds of ASSR were 80 dB nHL at 500 Hz, 90 dB nHL at 1 kHz, 100 dB nHL at 2 kHz, and 100 dB nHL at 4 kHz (right ear) and 90 dB nHL at 500 Hz, 60 dB nHL at 1 kHz, 110 dB nHL at 2 kHz, and 110 dB nHL at 4 kHz (left ear) (Figure 1(b)). The wave V thresholds of ABR of both ears were 90 dB, and DPOAE were absent bilaterally (Figure 1(c)). Tympanogram indicated almost normal function of the middle ear. The temporal bone CT scan suggested the proband without any malformation of middle or inner ear.

#### 3.1.2. Family 2

The proband II-1 of Family 2 suffered from profound hearing loss, which was shown by the auditory examination. The wave V thresholds of ABR of both ears were not extracted in 105 dB. The thresholds of ASSR were all 100 dB nHL at every frequency (Figure 1(e)) of each ear, and bilateral DPOAE were absent (Figure 1(f)). Tympanogram revealed a type A curve indicating a normal function of the middle ear. Temporal bone CT scans and MRI showed no obvious abnormalities. There was no family history of congenital deafness and no underlying environmental causes of hearing loss. The detailed clinical information is summarized in Table 1.

### 3.2. Mutation Identification and Analysis

**Figure 2:**
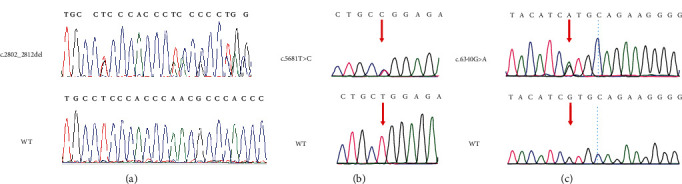
Sanger sequencing results of the c.2802_2812del (a), c.5681T>C (b), and c.6340G>A (c) mutations in the family members. Red arrows: sites of nucleotide changes.

Using deafness panel sequencing, 159 loci of 22 genes that cause congenital deafness were excluded. Whole-exome sequencing results were compared with the human reference genome (GRCh37/hg19). We found novel compound heterozygous mutation c.2802_2812del/c.5681T>C in Family 1 and c.5681T>C/c.6340G>A in Family 2. In Family 1, the c.2802_2812del mutation, which was located in exon 2 and passed on from her clinically normal father (Figure 2(a)), led to a frameshifting change in the N-terminal domain (p.Gln937Leufs∗39) and truncate mRNA translation resulting in lack of complete amino acid sequence. Occurring in exon 24, the c.5681T>C mutation, which was inherited from the unaffected mother (Figure 2(b)), led to a single substitution from leucine to proline at amino acid position 1894 in the ATPase motor domain (p.Leu1894Pro). Both of them were predicted to be deleterious variants based on the result of computer algorithms PolyPhen-2 and PROVEAN (Table 2), and these two variants were not seen in public databases dbSNP, 1000 Genomes Project, and ExAC. According to the American College of Medical Genetics and Genomics–Association for Molecular Pathology (ACMG–AMP) guidelines, the variant c.2802_2812del and c.5681T>C were classified pathogenic (PSV1+PM2+PM3+PM4+PP3) and likely pathogenic (PM2+PM3+PP3), respectively.

Interestingly, the variant c.5681T>C (p.Leu1894Pro) was also found in Family 2 and his unaffected father, and another variant was a reported variant c.6340G>A (p.Val2114Met) in exon 30, which was also detected in his mother (Figure 2(c)). The mutation c.6340G>A of the *MYO15A* gene, a known pathogenic missense mutation, leads to an alternation of a Valine with a Methionine at amino acid position 2114 in the MyTH4 domain. The c.6340G>A mutation was previously reported in another Chinese family and in two Egyptian families which is predicted to weaken the function of MyTH4 [15, 16]. It was predicted as deleterious by computational tools. Following the ACMG guidelines, the c.6340G>A variant was classified as pathogenic (PVS1+PM2+PM3+PP1+PP3). Detailed information of variants is shown in Table 2.

### 3.3. Functional Analysis of the Mutant Protein

**Figure 3:**
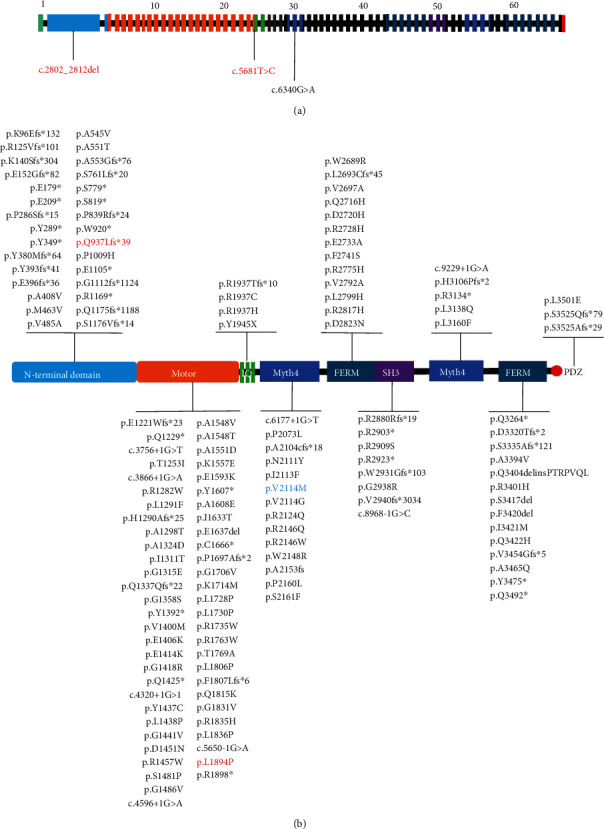
(a) Schematic diagram of 66 exons of the *MYO15A* gene is shown with all pathogenic mutations (arrows) of two families. Novel compound heterozygous *MYO15A* mutations are indicated in red. Previously reported mutation is indicated in black. (b) Overview of the reported *MYO15A* variants and their locations in the protein structure. The red words indicate the novel mutations, and the blue one refers to the reported variant that was detected in the proband in this study.

**Figure 4:**
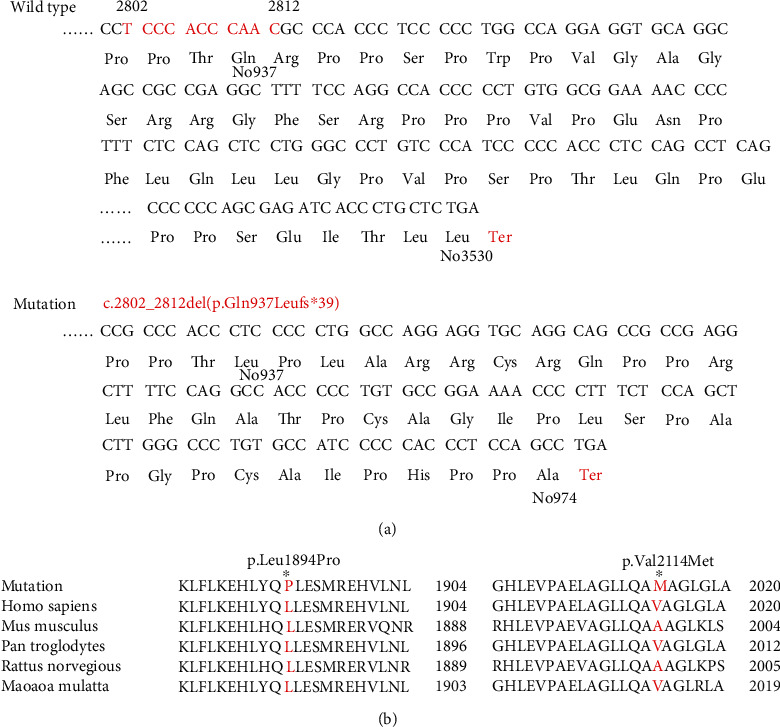
(a) Codon and amino acid coding diagram of the variant c.2802_2812del (p.Gln937Leufs∗39) of the proband II-1 of Family 1. The red letters indicate the changed amino acids and the site of the stop codon. In the mutant, no. 975 amino acid was converted into a termination codon. (b) Evolutionary conservation of no. 1894 and no. 2114 amino acid (red). Mutated site is indicated by an asterisk.

Myosin-XV differs from other myosin proteins in that it has a long N-terminal extending in front of the conserved motor region. It includes a N-terminal domain encoded by giant exon 2 (Figure 3(a)), the ATPase motor domain, a lever arm that consists of three IQ motifs, and a globular tail domain that contains MyTH4, FERM, SH3, and PDZ ligand (Figure 3(b)). The locations of the mutations in this study have been shown in Figure 4. The mutation c.2802_2812del identified in the proband II-1 of Family 1 results in a frameshift, and no. 975 amino acid was converted into a termination codon, which generated a truncated protein (Figure 4(a)). The evolutionary conservation result proved that the amino acid residues at the mutation sites were highly conserved among multiple species (Figure 4(b)). This strongly demonstrates the importance of these residues for the normal function of the protein.

## 4. Discussion

Hair cells (HCs) in the inner ear contribute to transducing sound waves into electric signals [17–21]. Hearing loss is one of the major disabilities worldwide, which is often induced by irreversible loss of sensory HCs and degeneration of the spiral ganglion neurons. Congenital hearing loss could be caused by genetic factors, cochlear infections, ototoxic drugs, and noise exposure, and genetic factors account for more than 60% of congenital hearing loss [22]. Each cochlear hair cell has a bundle of actin-based stereocilia that detect sound. The *MYO*15A gene encodes an unconventional myosin that is expressed in the cochlea, which traffics and delivers critical molecules required for stereocilia development and thus is essential for building the mechanosensory hair bundle [7, 23, 24]. Mouse models' studies show that *MYO15A* mutations can lead to abnormal hearing function caused by short stereocilia and by loss of the normal staircase structure of stereocilia in hair cells [25, 26].

The mutation c.2802_2812del results in a truncated protein caused by the frameshift. Like many previously reported pathogenic truncating mutations in the *MYO15A* gene, the c.2802_2812del variant is predicted to result in a truncated protein product without motor, IQ, MyTH4, FERM, SH3, and PDZ domains. Since DFNB3 is an autosomal recessive disorder and the proband's father is a heterozygous carrier, we strongly speculate that the variant c.2802_2812del might cause hearing loss related to the incomplete protein structure. A previous study indicated that pathogenic mutations that reside in the N-terminal domain are associated with a variety of mild hearing loss phenotypes [27–29]. In combination with other studies [30], our study suggests that a truncated mutation c.2802_2812del in the N-terminal domain of the *MYO15A* gene may contribute to a severe phenotype. These evidences indicate the diversity of auditory phenotypes due to *MYO15A* variation [31].

The c.5681T>C mutation leads to a single substitution from leucine to proline at amino acid position 1894. This variant is located in the motor domain of the *MYO15A* gene. Our literature review illustrated that the motion domain is a hot region affected by *MYO15A* variation and more and more variants were detected in this domain [32, 33]. This region contains actin and ATP binding sites that generate the force to transport actin filaments in vitro [34]. It is reasonable that motor domain dysfunction will lead to abnormal stereocilia associated with a severe deafness phenotype. A recent mechanism study revealed that the myosin-XV motor domain may exhibit strain sensitivity, suggesting that it could also act as a force-sensitive element bridging the membrane and actin cytoskeleton at the stereocilia tip which, in turn, makes a significant influence on human deafness of *MYO15A* mutation [24]. Profound hearing loss and null DPAOE response may be caused by this novel compound heterozygous mutations of the *MYO15A* gene. The c.6340G>A mutation is a known disease mutation (https://www.ncbi.nlm.nih.gov/snp/rs377385081) which was first reported by Yang et al. in 2013 [15]. In Yang's study, the biallelic mutations c.6340G>A/c.6956+9C>G were also found in the proband's deaf relatives for which they were identified as disease mutations. The c.6340G>A mutation is a missense mutation resulting in an alternation of a Valine with a Methionine at amino acid position 2114 in the first MyTH4 domain in myosin-XV. The MyTH4 domain of myosin has some roles in microtubule as well as actin binding at the plasma membrane. Mutations in this domain can disrupt the protein-protein interaction which is important for the normal function of hearing [35, 36].

Here, we found c.5681T>C was compound heterozygous with c.2802_2812del and c.6340G>A in these two irrelevant pedigrees. We concluded the c.5681T>C variant to be pathogenic for several reasons: (a) the amino acid residues at the mutation sites were highly conserved; (b) several pathogenic mutations have been found near this location; (c) detected in 2 sporadic pedigrees with similar DFNB3 phenotype; (d) the *MYO15A* gene is highly heterogeneous, and c.6340G>A mutation is a known disease mutation, which combined with c.5681T>C causing deafness. Moreover, both the 2 novel mutations were cosegregated with the profound deafness and were predicted to be pathogenic mutations by computer algorithms. These all suggested that c.5681T>C is a relevant and pathogenic mutation in the *MYO15A* gene responsible for the profound hearing loss of the probands.

In conclusion, our study demonstrated that two compound heterozygous mutations in the *MYO15A* gene (c.2802_2812del/c.5681T>C and c.5681T>C/c.6340G>A) were the pathogenic variants in two ARNSHL families. The c.2802_2812del and c.5681T>C mutations are reported for the first time, and both were strongly speculated as pathogenic mutations in the *MYO15A* gene. Our finding further extends the *MYO15A* gene mutation spectrum and enriches our knowledge of genotype-phenotype correlation in *MYO15A*-related deafness.

## Figures and Tables

**Table 1:** The clinical information of the patients.

Proband number	Age	Gender	Age onset	Hearing impairment	ABR	DPOAE	Tympanogram	MRI/CT
1	2 yr	F	0	Profound	85 dB	Absent	As(L)/A(R)	Normal
2	1 yr	M	0	Profound	105 dB	Absent	A(L)/A(R)	Normal

ABR: auditory brainstem response; DPOAE: distortion product otoacoustic emissions; A: A type; As: As type.

**Table 2:** Identified pathogenic variants in the MYO15A gene in this study and their prediction results by computer algorithms.

Nucleotide change	Type of variation	Gene subregion	Amino acid change	MutationTaster^a^	PROVEAN^b^	PolyPhen-2^c^	Novelty
c.2802_2812del	Truncation	Exon 2	p.Gln937Leufs∗39	DC	Deleterious (score -65.157)	—	Novel
c.5681T>C	Missense	Exon 24	p.Leu1894Pro	DC	Deleterious (score -6.817)	Probably damaging (score 0.988)	Novel
c.6340G>A	Missense	Exon 30	p.Val2114Met	DC	Deleterious (score -2.696)	Probably damaging (score 0.982)	[15, 16]

^a^DC: disease causing; PO: polymorphism. ^b^The PROVEAN scores indicated deleterious and neutral function, respectively, with a cut-off score set at -2.5. Variants with a score equal to or below -2.5 are considered “deleterious”; variants with a score above -2.5 are considered “neutral.” ^c^The PolyPhen-2 score ranges from 0.0 to 1.0. Variants with scores of 0.0 are predicted to be benign. Values closer to 1.0 are more confidently predicted to be deleterious. The score can be interpreted as follows: 0.0 to 0.15: benign; 0.15 to 1.0: possibly damaging; 0.85 to 1.0: probably damaging.

## Data Availability

The data which supports the conclusions of our study is available upon request.
